# Disruption of fish gut microbiota composition and holobiont’s metabolome during a simulated *Microcystis aeruginosa* (Cyanobacteria) bloom

**DOI:** 10.1186/s40168-023-01558-2

**Published:** 2023-05-16

**Authors:** Alison Gallet, Sébastien Halary, Charlotte Duval, Hélène Huet, Sébastien Duperron, Benjamin Marie

**Affiliations:** 1 UMR7245 Molécules de Communication et Adaptation des Micro-organismes, Muséum National d’Histoire Naturelle, CNRS, Paris, France; 2 UMR1161 Virologie, École Nationale Vétérinaire d’Alfort, INRA - ANSES - ENVA, Maisons-Alfort, France; 3 Institut Universitaire de France, Paris, France

**Keywords:** Cyanobacteria, Gut microbiota, Metagenomics, Metabolomics, Medaka fish

## Abstract

**Background:**

Cyanobacterial blooms are one of the most common stressors encountered by metazoans living in freshwater lentic systems such as lakes and ponds. Blooms reportedly impair fish health, notably through oxygen depletion and production of bioactive compounds including cyanotoxins. However, in the times of the “microbiome revolution”, it is surprising that so little is still known regarding the influence of blooms on fish microbiota. In this study, an experimental approach is used to demonstrate that blooms affect fish microbiome composition and functions, as well as the metabolome of holobionts. To this end, the model teleost *Oryzias latipes* is exposed to simulated *Microcystis aeruginosa* blooms of various intensities in a microcosm setting, and the response of bacterial gut communities is evaluated in terms of composition and metabolome profiling. Metagenome-encoded functions are compared after 28 days between control individuals and those exposed to highest bloom level.

**Results:**

The gut bacterial community of *O. latipes* exhibits marked responses to the presence of *M. aeruginosa* blooms in a dose-dependent manner. Notably, abundant gut-associated Firmicutes almost disappear, while potential opportunists increase. The holobiont’s gut metabolome displays major changes, while functions encoded in the metagenome of bacterial partners are more marginally affected. Bacterial communities tend to return to original composition after the end of the bloom and remain sensitive in case of a second bloom, reflecting a highly reactive gut community.

**Conclusion:**

Gut-associated bacterial communities and holobiont functioning are affected by both short and long exposure to *M. aeruginosa*, and show evidence of post-bloom resilience. These findings point to the significance of bloom events to fish health and fitness, including survival and reproduction, through microbiome-related effects. In the context of increasingly frequent and intense blooms worldwide, potential outcomes relevant to conservation biology as well as aquaculture warrant further investigation.

Video Abstract

**Supplementary Information:**

The online version contains supplementary material available at 10.1186/s40168-023-01558-2.

## Background

Organisms living in lakes and ponds are exposed to cyanobacterial blooms throughout their life [1–4]. Blooms are natural events, yet increased eutrophication and global change associated with human activities make them increasingly frequent, abundant and persistent worldwide [5, 6]. Cyanobacterial blooms affect the whole ecosystem, including the health of teleost fish which occupy higher trophic levels [1, 2, 7]. They can trigger mass mortalities of animals due to oxygen depletion. Cyanobacterial metabolites display a broad range of bioactivities and include various toxins, digestive enzyme inhibitors, antimicrobials, and cytotoxic compounds [8]. As a consequence, cyanobacteria cells, extracts and purified cyanotoxins all induce deleterious effects on teleost fishes [2, 9–12]. One of the most toxic and frequent cyanotoxins is microcystin-LR (MC-LR), a hepatotoxin that accumulates in fish liver mostly following ingestion of contaminated prey or water. Intraperitoneal injection doses of 400 to 1000 μg.kg^−1^ are lethal to fish [13, 14]. MC-LR adversely affects fish development, reproduction and behavior [15, 16]. It specifically affects the liver in which MC-LR triggers inflammation, oxidative stress, and tissue necrosis [17–19]. Aquatic animals are exposed to cyanobacteria and their toxins through oral ingestion and transfer absorption through the intestine [15, 20, 21]. In this respect, the role of gut-associated microbiota in holobiont’s response is currently underestimated [22]. In teleosts, gut microbiome tends to be less diverse than observed in larger vertebrates, and is often dominated by Proteobacteria, Bacteroidetes, and Firmicutes [23, 24]. As for other vertebrates, gut-associated symbionts play important roles in nutrition (polymer degradation, vitamin production), immunity, protection against pathogens, and homeostasis [25–27]. Various host- and environment-related factors are reported to influence gut microbiome structure [28], and the gut microbiota has recently emerged as a primary target for microbiome-aware ecotoxicological concerns [29–33]. However, only a limited number of studies have investigated the effect of cyanobacterial blooms on fish gut microbiota [10, 34–36]. Whole chemical extracts of few *Microcystis* strains were for example shown to influence the composition of gut bacterial communities of medaka fish in microcosm-based experiments, while MC-LR alone did not [35]. This and few other works emphasize that metabolite cocktails and whole cells, rather than toxins alone (microcystins), should be considered for realistic assessment of the microbiome impairs [2, 15, 35, 37], yet the effect of exposure to a range of environmentally relevant levels of cyanobacteria has not been evaluated so far.

In the present study, the impact of a cyanobacterial bloom on the composition and functions of fish gut-associated bacterial communities, and on the metabolite composition in various host tissues is evaluated using an experimental approach. Due to its resistance to stress and disease, the teleost fish *Oryzias latipes* (medaka) has emerged as a model of choice for aquatic ecotoxicology in general, and a body of literature now specifically documents the effects of cyanotoxins on animal tissue [38–41]. Herein, *O. latipes*, has been exposed to *Microcystis aeruginosa*, the most common bloom-forming cyanobacterium in temperate lentic freshwaters [42]. Three exposure levels were defined within a range of values commonly reported in natural lakes and ponds. The highest exposure level, 100 µg.L^−1^ Chl*a*, corresponds to a high bloom intensity, yet commonly reported in eutrophic lakes [43], while the medium level (10 µg.L^−1^ Chl*a*) is commonly considered a threshold value indicative of a bloom [44]. A first 28-day exposure simulated a long bloom event. Because blooms are highly dynamic events in natural systems, post-bloom resilience was investigated. Then, the hypothesis of a priming effect, translating into a lower impact of a second bloom, was tested. To this end, a post-bloom depuration phase was conducted for 4 days, followed by a second exposure to the highest *M. aeruginosa* concentration for 5 days. Bacterial community compositions were characterized using 16S rRNA gene sequencing, and metabolite contents were profiled by LC-MS/MS. Metagenomes of unexposed control fish gut communities were compared to those of specimens exposed to the highest bloom level to compare their respective annotated functions. Bacterial community and metabolites compositions were then compared using a multi-omics approach to identify correlation networks associated with teleost holobiont response. By testing the dose-dependent effect of a cyanobacterial bloom on teleost gut bacterial microbiota, documenting holobiont post-bloom response, and investigating the effect of a second bloom, this study addresses for the first time the dynamics of holobiont response to cyanobacterial blooms.

## Methods

### Experimental design and sampling

Experimental procedures were carried out in accordance with European legislation on animal experimentation (European Union Directive 2010/63/EU) and were approved for ethical contentment by an independent ethical council (CEEA Cuvier n°68) and authorized by the French government under reference number APAFiS#19316-2019032913284201 v1.

**Fig. 1:**
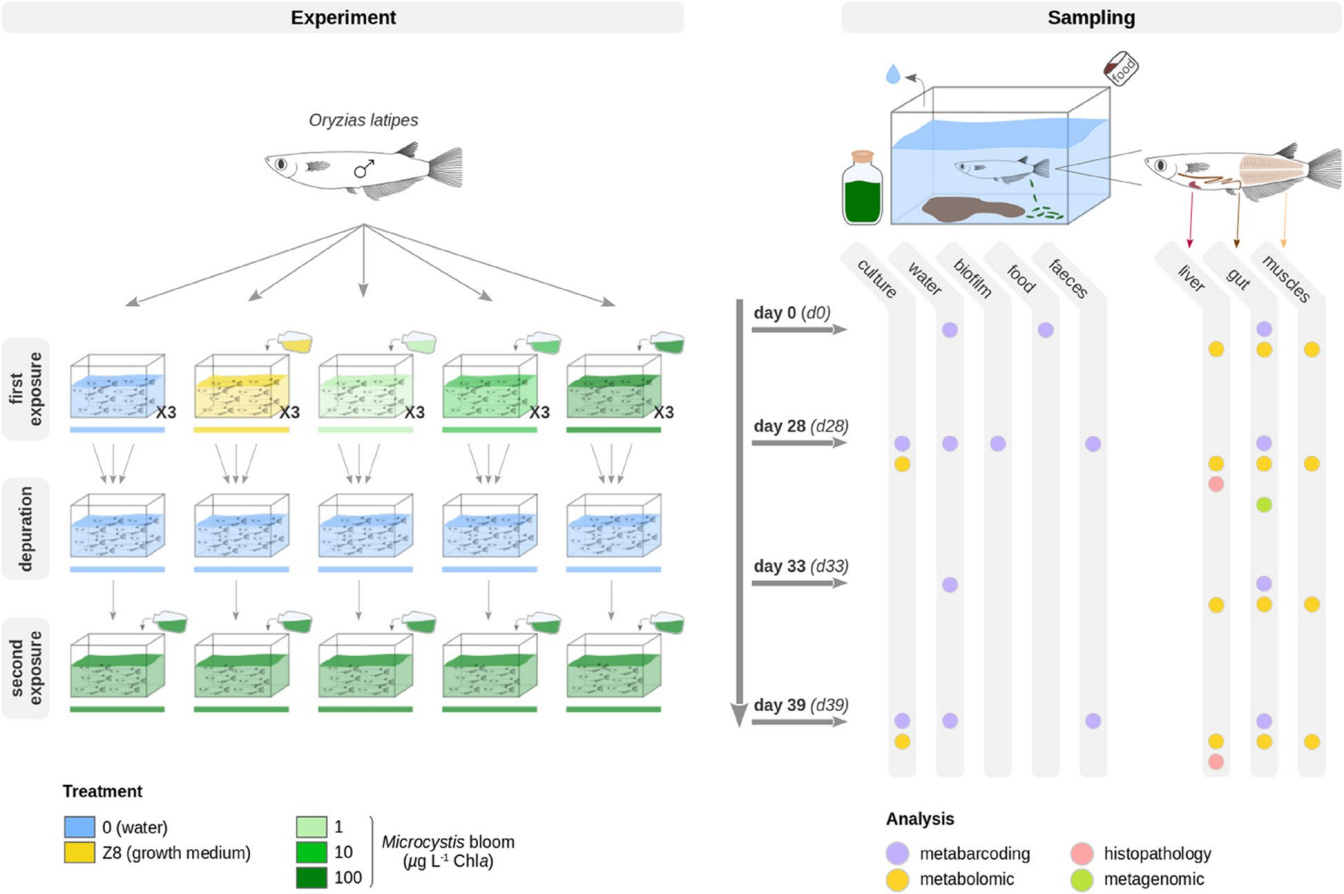
Experimental design and *O. latipes* sampling strategy. After a 14-day acclimatization period, adult male *O. latipes* were exposed to two successive exposures. During the first 28 days (d0 to d28), fish were exposed to five different treatments, each carried out in three 10-L aquaria. Treatments consisted of two controls (0, Z8) and three concentrations of *Microcystis aeruginosa* (1, 10, and 100 µg.L^−1^ Chl*a*). After that, a 4-day depuration phase followed (d28 to d33) performed in clear water (0), then fish were exposed again for 5 days (d33 to d39) to *M. aeruginosa* (100 µg.L^−1^ Chl*a*). Colors represents the different treatments: blue = water, yellow = Z8 growth medium, light green = 1 µg.L^−1^ Chl*a*, green = 10 µg.L^−1^ Chl*a*, dark green = 100 µg.L^−1^ Chl*a*. Samples were collected at d0 (day 0), d28 (day 28), d33 (day 33), and d39 (day 39). Further analyses were performed on *M. aeruginosa* culture, water, biofilm, fish food, feces samples, and different tissues (Supplementary Note 1 and Figure S1a–d). Dataset S2 provides details on sampling counts

Experiments were performed in 10-liter aquaria (microcosms) with 7-month-old adult male Japanese medaka fish *Oryzias latipes* provided by the AMAGEN platform (Gif-sur-Yvette, France). Before the whole experiment, five fish were sampled as controls for the histological analyses then fish were pre-acclimatized in clear water (2 weeks) in 15 aquaria, each one containing 8 fish. Prior to the first exposure at day 0, five fish were randomly sampled among the aquaria, and 10 mL of water of each aquarium were pooled, as references of initial fish and water conditions (d0, Fig. 1). Fish were then exposed for 28 days to five treatments: water (control, 0); water containing Z8 medium [45], *i.e.*, the medium used to cultivate cyanobacteria (control for water enrichment with nutrients from Z8); and water containing three environmentally relevant concentrations of live *Microcystis aeruginosa*, *i.e.*, 1, 10, and 100 µg.L^−1^ Chl*a* (1, 10, 100), respectively (d28, Fig. 1). Each treatment was carried out in three aquaria (labelled a, b, and c). At d28, four fish, and 150 mL of water were sampled in each aquarium. Bottom-growing biofilms and faeces were also sampled by scraping using sterile scalpel and tweezers, respectively. After sampling, remaining fish from each of the three aquaria exposed to one condition were pooled and transferred to a single aquarium, filled with clear water (treatment 0) for a 4-day depuration period of exactly 110 h (d33, Fig. 1). At d33, three fish and 150 mL of water were sampled in each aquarium. All fish were then exposed in the same aquaria for 5 days (exactly 134 h) to the highest concentration of *M. aeruginosa*, 100 µg.L^−1^ Chl*a* (d39, Fig. 1). At d39, four fish, 150 mL of water and faeces were sampled in each aquarium. Along the two successive exposures, 10 mL of *M. aeruginosa* culture were sampled every 2 days but only one sample per week was analyzed further. Dataset S1 provides details of sampled individuals and performed analyses.

### *Microcystis aeruginosa* production

Blooms were simulated in lab using the non-axenic and easy-to-cultivate *M. aeruginosa* mono-clonal strain PMC 728.11 maintained in the Paris Museum Collection which can produce diverse variants of bioactive metabolites (Supplementary Note 2). The strain was cultivated in Z8 medium at 25 ± 1 °C with a 16-h:8-h light/dark cycle (at 14 µmol.m^−2^.s^−1^) in 2-L bottles all along the experiment (LED strips TR-LD-24VDC-SMD5050-NW and modulator). The concentrations of *M. aeruginosa* were estimated using Chlorophyll *a* extraction [46] and absorbance measurements as a proxy using a spectrophotometer (Cary 60 UV-Vis, Agilent). Every 2 days, after water renewal, *M. aeruginosa* was measured in each aquarium using a fluorometer (FluoroProbe III, bbe Moldaenke), and appropriate volume of the culture was added as per the desired final concentrations (1, 10, or 100 µg.L^−1^ Chl*a*).

### Monitoring of experimental parameters

Water was aerated using a pump (Air-Flow 4, SuperFish, Netherlands). Every second day throughout the experiment, water parameters were monitored (pH, temperature, conductivity, nitrates, and nitrites), aquaria were cleaned (faeces removed by aspiration), half of the water was replaced with fresh water composed of 2/3 osmosis (RiOs 5, Merck Millipore) and 1/3 filtered. On the same timeline, *M. aeruginosa* concentrations were measured and adjusted to maintain bloom levels in exposed treatments, and 1 mL of sterile Z8 medium was added in the Z8 treatment. Fish were exposed to constant temperature (23 ± 1 °C), pH (7.5 ± 0.1), and conductivity (234 ± 22 µS.cm^−1^), to low levels of nitrates (≤ 1 mg.L^−1^) and nitrites (≤ 4 mg.L^−1^), to a 12-h:12-h light/dark cycle (Philips MASTER TL-D 36W/840). They were fed twice daily (~ 3–5% of the fish biomass per day) with Nutra HP 0.3 (Crude protein 57, Crude fat 17, N.F.E 7.5, Ash 10, Crude fiber 0.5, Phosphorus 1.7, Vitamins A, D3, E; Skretting, Norway). Microcystin (MC) concentration was monitored on a regular basis on water samples in *M. aeruginosa*-containing treatments and quantified using enzyme-linked immunosorbent assay (ELISA) analyses (Microcystins-ADDA SAES ELISA, Eurofins Abraxis). Each sample was analyzed in duplicates and MC concentrations were determined according to the MC-LR response standard curve (Dataset S2).

### Fish, *M. aeruginosa* culture, water, biofilm, food, and feces processing

Fish were anesthetized in 0.1% tricaine methanesulfonate (MS-222; Sigma, St. Louis, MO) buffered with 0.1% NaHCO_3_ and sacrificed. Whole guts (including content, due to small size), muscles and livers were dissected, flash-frozen in liquid nitrogen and stored at − 80 °C. For histopathological examinations, livers from fish sampled before the whole experiment and at d28 and d39 (one fish per aquarium) were dissected, fixed in Davidson fixative as previously described [15], maintained for 24 h at 4 °C then dehydrated in 70% ethanol and conserved at 4 °C. Livers samples were then embedded in paraffin and blocks were cut into 4-µm thick sections, stained with hematoxylin-eosin-saffron (HES), periodic acid schiff (PAS), and Perls Prussian blue, and observed under photonic microscope (Zeiss, Germany). Aquarium water samples were filtered on a 0.22-µm filter (Nucleopore Track-Etch Membrane) and frozen. *M. aeruginosa* culture (4 mL) and biofilm samples were centrifugated (10 min, 10 °C, 3220×*g*) and pellets were frozen. Feces pellets were directly frozen. A food sample was kept for DNA extraction.

### Metabolites extraction

Metabolite contents were extracted from fish livers, whole guts (including content) and muscles, *M. aeruginosa* cultures, and biofilms. Prior to sonication, muscles were freeze-dried then ground using a bead beater (TissueLyser II, Qiagen) while cultures and biofilms were only freeze-dried. Samples were weighted, then sonicated in 75% methanol (1 mL per 100 mg of tissue, 3×, on ice) and centrifuged (10 min, 4 °C, 15,300×*g*). Supernatants containing metabolite extracts were kept at – 20 °C for mass spectrometry analyses. All pellets were discarded, except gut pellets dried and kept at – 80 °C to perform a subsequent DNA extraction on the same gut tissue.

### Mass spectrometry data processing and analysis

Each metabolite extract from fish livers, guts and muscles was analyzed by ultra high-performance liquid chromatography (UHPLC; ELUTE, Bruker) coupled with a high-resolution mass spectrometer (ESI-Qq-TOF Compact, Bruker) at 2 Hz speed, on simple MS mode then on broad-band Collision Ion Dissociation (bbCID) or autoMS/MS mode on the 50–1500 *m*/*z* range. Three feature peak lists were generated from MS spectra within a retention time window of 1–15 min and a filtering of 5000 counts using MetaboScape 4.0 software (Bruker). The three peak lists consisted of the area-under-the-peaks of extracted analytes from the three tissues (gut, liver, muscle) sampled at d28, d33, and d39, resulting in 1672, 909, and 3127 analytes, respectively. The genuine metabolite content of the culture was investigated on metabolite extracts using LC-MS/MS approach, combined with molecular network analysis and metabolite annotation using a cyanobacterial metabolite reference database, as previously described [47]. Prior to analyses, Pareto scaling was applied on the datasets [48]. Principal component analyses (PCA) were performed to compare the metabolite composition among groups using the *mixOmics* package [49] in R 4.1.0 (R Core Team, 2021). The variance among groups was compared conducting PERMANOVA (999 permutations) based on euclidean distance with *vegan* [50] followed by Bonferroni-adjusted pairwise comparisons with *RVAideMemoire* [51].

### DNA extraction

DNA was extracted from gut pellets resulting from the metabolites extraction, culture, biofilm, faeces, water filters and food using the ZymoBIOMICS DNA Miniprep kit (Zymo Research, California). Prior to DNA extraction, all pellets were re-suspended in Eppendorf tubes with 750 µL of the ZymoBIOMICS^TM^ lysis solution, then the contents were transferred to the ZR BashingBead^TM^ lysis tubes. Water filters were cut into pieces then transferred to the ZR BashingBead^TM^ lysis tubes. All steps were conducted following the manufacturer’s instructions except for mechanical lysis, achieved on a bead beater (TissueLyser II, Qiagen) during 6 × 1 min. An extraction blank was sequenced as a control and the 8 ASVs that were > 1% of reads were removed from the whole dataset as potential contaminants. Together, they represented 0 to 2.8% of reads in the different samples (mean 0.46%). The quality and quantity of the extracted DNA was tested on Q-bit (Thermo).

### Bacterial 16S rRNA gene sequencing and analyses

The V4–V5 variable region of the 16S rRNA gene was amplified using 479F (5′-CAGCMGCYGCNGTAANAC-3′) and 888R primers (5′-CCGYCAATTCMTTTRAGT-3′) [52], and sequenced (Illumina MiSeq paired-end, 2 × 250 bp, GenoScreen, France). Paired-end reads were demultiplexed, quality controlled, trimmed and assembled with FLASH [53]. Sequence analysis was performed using the QIIME 2 2020.11 pipeline [54]. Chimeras were removed and sequences were trimmed to 367 pb then denoised using the *DADA2* plugin, resulting in Amplicon Sequence Variants (ASVs) [55]. ASVs were affiliated to taxa using the SILVA database release 138 [56] using the *feature-classifier* plugin and *classify-sklearn* module [57, 58]. Sequences assigned as Eukaryota, Archaea, Mitochondria, Chloroplast and unassigned were removed from the dataset then the sample dataset was rarefied to a list of 6978 sequences.

Alpha- and beta-diversity analyses were performed using the *phyloseq* [59], *vegan* and *RVAideMemoire* packages in R. Linear mixed models (LMMs) were used to compare species richness among the five treatments and the three replicate aquaria within each treatment at d28, using the *MuMIn* [60] and *lmerTest* [61] R packages. We adapted the LMMs to the non-independency of individuals within each replicate, and defined the replicates as random effects and the five treatments as fixed effects, according to the formula Y ~ treatment + (1 | treatment : aquarium). Principal coordinates analyses (PCoA) based on weighted and unweighted UniFrac distances were performed to examine the dissimilarity of bacterial composition between groups. Among- and within-group variance levels were compared using PERMANOVA (999 permutations) and PERMDISP (999 permutations), respectively. Differentially abundant taxa across groups were identified using the linear discriminant analysis (LDA) effect size (LEfSe) tool [62] in the Galaxy workspace [63] (http://huttenhower.sph.harvard.edu/lefse/). Default parameters were applied using a LDA score threshold of 3.5 and the multi-class strategy (one-against-all).

### Microbiome-metabolome integrative analysis

The integration of datasets, *i.e.*, the area-under-the-peaks in metabolite profiles and the ASV counts describing the bacterial communities in the same sample, was performed using the *mixOmics* package in R. Pareto scaling was applied on the metabolome data, and a centered log-ratio transformation then a pre-filtering keeping only abundant ASVs, (*i.e.*, representing at least 1% of the reads in at least one sample), were applied on the microbiome data. Following unsupervised analyses on each dataset, completed to explore and visualize any similar changes according to treatments, the integration was carried out. A supervised Projection to Latent Structures Discriminant Analysis (PLS-DA) was performed using DIABLO (Data Integration Analysis for Biomarker discovery using Latent cOmponent) [64], enabling to identify highly-correlated variables (metabolites and ASVs) also discriminating the different treatments. The integration of both datasets was realized using the full-weighted design matrice and the *block.plsda* function implemented in *mixOmics*. The *plotDiablo* function enabled to check the well maximized covariation between datasets by displaying a Pearson correlation score. Then, relevance networks displaying the most discriminant covariates (metabolites, ASVs) were produced using the *network* function with Pearson correlation cut-offs [65].

### Metagenomic sequencing and analysis

Shotgun metagenome sequencing was performed on DNA from 10 gut samples collected at d28, 5 in treatment d28_0 and 5 from d28_100 (Illumina HiSeq, 2 × 150 bp, GenoScreen, France). Reads corresponding to animal sequences were identified by aligning each dataset against *Oryzias latipes* available at the NCBI, using BBMap/bbsplit [66], and discarded. Remaining reads from each sample were assembled using metaSPAdes with default parameters [67]. Scaffolds were first taxonomically annotated using Contig Annotation Tool (CAT) [68] and Kaiju [69] allowing to detect sequences from *O. latipes* retroviruses and *Microcystis* genome which were then discarded. All scaffolds were clustered using MyCC [70] (k-mer size = 4, minimal sequence size = 1000) and bins were taxonomically annotated using Bin Annotation Tool [68]. Completeness of bins was assessed using CheckM [71]. Relative abundance of bins in each sample were also determined using BBMap. Significant bins between the two treatments were determined using Wilcoxon rank-sum test. Finally, coding sequences predicted by Prodigal [72] were functionally annotated using eggNOG-emapper [73]. Resulting KEGG annotations were used as input to MinPath [74] in order to obtain the MetaCyc pathway information, and to Omixer-RPM [75] to specifically characterize gut metabolic modules (GMMs) as firstly described in [76], with pathway coverage threshold ≥ 80%.

## Results

### Monitoring of experiments

*Oryzias latipes* fish were maintained in suitable and stable conditions, and no fish died during the whole experimentation (Fig. 1 and Dataset S2). During the first 28 days long exposure (d0–d28), concentrations of *Microcystis aeruginosa* in the three treatments (d28_1, d28_10, and d28_100) corresponded to expected levels, 1.0 ± 0.2, 10.0 ± 0.8, and 100.2 ± 7.8 µg.L^−1^ Chl*a*, respectively. Microcystin levels in water were 0.4 ± 0.0 and 10.4 ± 2.1 µg MC-LR eq.L^−1^ in the d28_10 and d28_100 treatments, respectively, while microcystin was below detection level (< 0.15 µg.L^−1^) in d28_1. During the second *M. aeruginosa* exposure (d33–d39), fish were exposed to 102.8 ± 2.9 µg.L^−1^ Chl*a* and 11.2 ± 2.8 µg MC-LR eq.L^−1^. The metabolic content of *Microcystis aeruginosa* cultures was examined (see Supplementary Note 2, Dataset S3, and Table S1). Histopathological analyses did not reveal noticeable visual differences in fish liver tissue, with little to no carbohydrate reserves, and no lipofuscin and macrophagic hemosiderin.

### Diversity and composition of the gut bacterial microbiota after 28 days of exposure

**Fig. 2:**
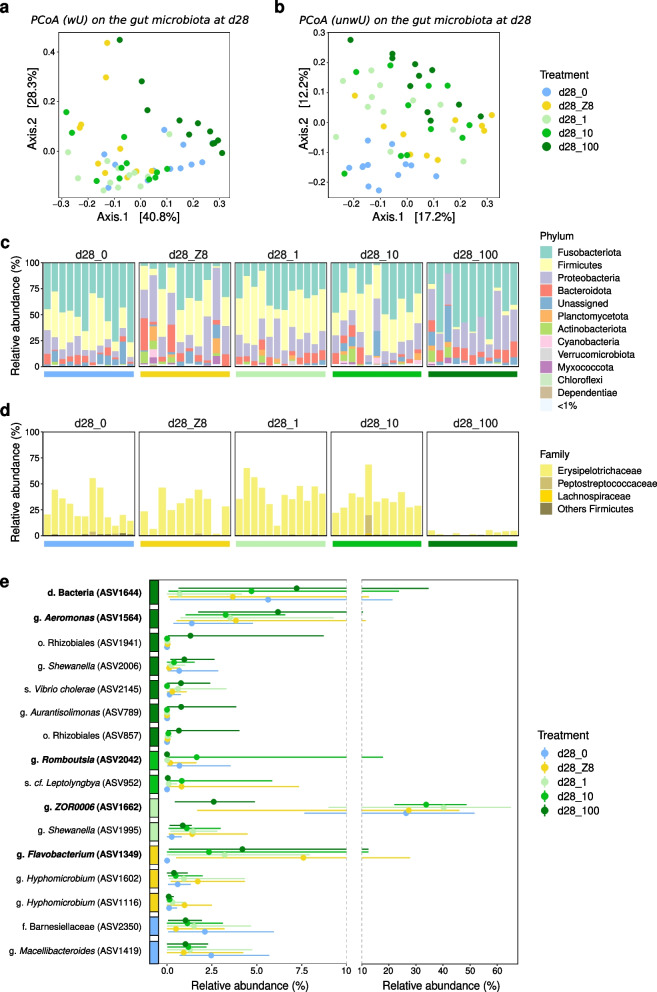
Changes in the composition of gut bacterial communities after 28 days of exposure. **a**, **b** PCoA using the weighted (**a**) or unweighted (**b**) UniFrac distances on fish gut bacterial community in the five treatments. **c** Relative abundance of bacterial phyla across treatments. **d** Relative abundance of Firmicutes members (family level). Firmicutes are mainly represented by the Erysipelotrichaceae family, and a single ASV (ASV1662). Colored horizontal bars represent the different treatments (see Fig. 1). **e** Significant ASVs from the linear discriminant analysis (LDA) effect size (LEfSe) with a LDA score above 3.5, and their relative abundances across d28 treatments. The colored boxes on the y-axis represent the treatment where each ASV is most abundant; dots represent the average relative abundance; line spreads over the range of observed values. Only dominant ASVs, *i.e.*, representing at least 10% of reads in at least one sample were further considered

At d28, gut communities display between 42 and 219 ASVs with higher average bacterial richness (136 ± 56 ASVs) and evenness (0.556) reported in fish guts exposed to d28_Z8, and lower average bacterial richness (82 ± 25 ASVs) and evenness (0.391) in the treatment d28_0 (Dataset S4). Gut-associated species richness is highest in the d28_Z8 treatment (LMM, *p* < 0.05), while no differences are observed among replicates within each treatment (LMM, *p* > 0.05). The Shannon index increases slightly with *Microcystis* concentration (d28_1: 2.09, d28_10: 2.14, d28_100: 2.28). Visual comparisons on individual plots of principal coordinates analyses (PCoA) based on the weighted or unweighted UniFrac distances suggest changes occur in terms of both abundances as well as community membership when fish are exposed to *M. aeruginosa* (d28_1, d28_10, d28_100) or d28_Z8 compared to d28_0 (Fig. 2a, b). Bacterial community composition appears different among treatments (PERMANOVA, weighted UniFrac, *p* < 0.001), notably between d28_100 and the other four treatments (*p* < 0.01), as well as between d28_0 and d28_Z8, d28_1, and d28_100 (*p* < 0.02). Differences between d28_Z8, d28_1, and d28_10 are not significant (*p* > 0.23) indicating comparable effect of a Z8 nutrients enrichment and lower *M. aeruginosa* doses. In addition, levels of variance in the different treatments are not significantly different (PERMDISP, *p* > 0.19).

In treatment d28_0 (water-exposed control), Fusobacteriota (50 ± 16%), Firmicutes (28 ± 15%), Proteobacteria (10 ± 6%), and Bacteroidota (5 ± 3%) dominate the microbiota, altogether representing 92.8 ± 6.9% of the reads. Relative abundances of these phyla are different among other treatments. Notably, Firmicutes are far less abundant in d28_100 (3 ± 2%) compared to other treatments (d28_0: 28 ± 15%, d28_Z8: 28 ± 15%, d28_1: 41 ± 14%, d28_10: 36 ± 12%) (Fig. 2c).

Many ASVs display significant differences across treatments in their relative abundances (Fig. 2e and Figure S2a). We focus on the six dominant ASVs, *i.e.,* representing at least 10% of reads in at least one sample, accounting from 11 to 72% of the reads. ASV1349, ASV1564, and ASV2363 exhibit lower relative abundances in d28_0 (average below 0.7%). ASV1349 (*Flavobacterium*) and ASV1564 (*Aeromonas*) are more abundant in d28_Z8 (7.6 ± 10% and 3.8 ± 3.5%, respectively) and d28_100 (4.2 ± 4.1% and 6.2 ± 2.9%, respectively). ASV2363 (*Reyranella*) is also more abundant in d28_100 (5.8 ± 11.1%). ASV1662 affiliated to the genus *ZOR0006*, is the main Firmicutes (96–100% of all Firmicutes reads), and as previously mentioned, is least abundant in d28_100 compared to other treatments (Fig. 2d). ASV2042 (*Romboutsia*), dominant in a single sample (17.8%) while below 3.5% in all others, was not further considered. Finally, ASV1644 (unassigned Bacteria) displays similar abundances in treatments d28_0 (5.6 ± 6.0%) and d28_100 (7.2 ± 9.7%) but is much less abundant in treatment d28_1 (0.7 ± 1.2%). Aside from the dominant ASVs that vary, ASV1620 (*Cetobacterium*) displays non-significant differences in abundance among treatments, representing 48 ± 16% of reads in d28_0 and average 27% to 46% of reads in other treatments.

### Metagenome-based comparison of gut communities in d28_0 and d28_100

Metagenome datasets obtained from 5 fish guts from d28_0 and 5 others from d28_100 yielded negligible amounts of Archaea and non-fish Eukaryota sequences. Among the 32 bacterial bins obtained, 8 were found to be more abundant after treatment (*p* < 0.05, Wilcoxon rank-sum test) (Figure S2b), including seven which vary concordantly with ASVs sharing the same taxonomic annotation (*Flavobacterium*, *Aeromonas*, *Gemmobacter*, Rhizobiales, Figure S2a, b). Three bins showed a decreasing abundance after treatment, including bin24, affiliated to the Firmicutes, with a majority of coding sequences associated to *ZOR0006* (58.06%), the genus corresponding to aforementioned ASV1662.

**Fig. 3:**
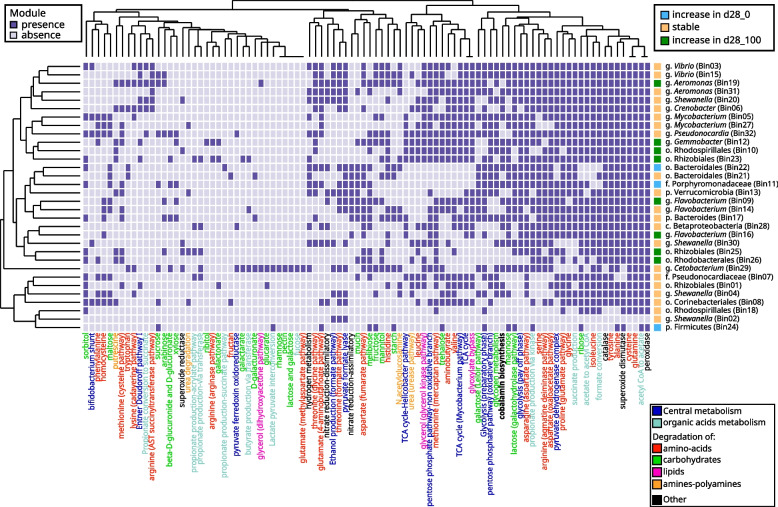
Heatmap of GMM presence/absence in bins obtained from the medaka microbiome. GMM label colors represent the metabolic categories. Bin label colors correspond to their abundance variation between experimental conditions. Dendrograms are based on hierarchical clustering

Gut Metabolic Modules were also investigated, with a total of 93 found within the 32 bins (Fig. 3). Each bin possesses a specific set of GMM involved in central metabolism and degradation of organic molecules or cobalamin (vitamin B_12_) biosynthesis. A hierarchical clustering based on the GMM presence/absence failed to gather bins following their abundance variation behavior between treatments. Interestingly, cobalamin biosynthesis is a largely shared metabolic feature in the medaka gut microbiome, since 17 bins harboured this GMM, including bin29 associated with *Cetobacter*. *ZOR0006* related bin24 carries a limited set of GMM mostly involved in Carbohydrates degradation. It also harbours the lactate pyruvate interconversion function that is only shared with the *Cetobacterium*-affiliated bin29. When considering genomic investment in the three major organic substrate types degradation, namely amino-acids, carbohydrates and lipids, bins were split into two subpopulations (Figure S3): a first group gathering bacteria with a generalist strategy (in the center of the ternary plot), and a second one that is highly specialized in carbohydrates degradation, the latter including the three bins showing an abundance decrease in exposure condition (bin11, bin24, and bin22). Beyond GMM, only 0.3% (6) of all enzymes are specific to these three bins (Figure S2c), including one involved in lactose degradation (KO2788) but not essential for this pathway. On the other hand, 4.3% (95) of enzymes are specifically found in the 8 bins that are enriched in d28_100 (namely bin9, bin10, bin12, bin16, bin19, bin23, bin25, bin26). These represent 95 specific enzymes, including 14 involved in the biosynthesis of secondary metabolites, 10 in porphyrin and chlorophyll metabolism, and 6 in the biosynthesis of cofactors. The analysis of clusters of orthologous groups (COGs), regardless of their annotation status, reveals that 963 of them (4.4% of the total COGs) are specific to the three bins that are the most abundant in d28_0, and that 2316 others (10.7% of the total gene families) are specific to the 8 bins that are the most abundant in d28_100. However, one should notice that most of these COGs carry no known functional annotation, compromising our understanding of the influence of gene content variations on microbiota functioning based on metagenomic assumption.

### Gut metabolome variation and integration with gut microbiota composition after 28 days of exposure

**Fig. 4:**
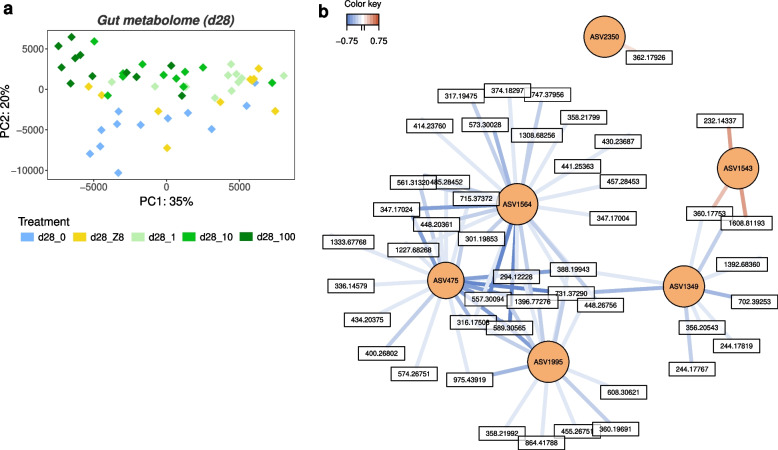
Modification of the gut metabolome composition associated with bacteria changes. **a** Principal component analysis (PCA) representing gut metabolite profiles at d28. **b** Relevance network analysis illustrating the most correlated metabolites (in white) and ASVs (in orange) discriminating among the five treatments. Only variables (ASVs, metabolites) with correlation values above ± 0.695 are displayed. Pearson correlations between covariate metabolites and ASVs are represented by coloured segments (blue: negative association, red: positive association)

A total of 1674 metabolites were detected across gut samples at d28. The PCA analysis separates the metabolite profiles of fish exposed to the different *M. aeruginosa* levels (d28_1, d28_10, d28_100) along the first axis, while the second axis mostly separates d28_0 from other treatments (Fig. 4a). The gut metabolite composition is different among treatments (PERMANOVA, euclidean distance, *p* < 0.001), between d28_0 and the three *M. aeruginosa* treatments (*p* = 0.01), and between d28_100 and all treatments (*p* < 0.03) but d28_10 (*p* = 0.19). Treatments d28_Z8, d28_1, and d28_10 do not display differences (*p* > 0.3). No significant variation is observed among treatments in livers (*p* = 0.13) (Figure S4a). In muscles, differences occur (*p* < 0.001) especially between d28_0 and both d28_Z8 and d28_1 (*p* = 0.01), and between d28_100 and all other treatments (*p* = 0.01) except d28_0 (*p* = 0.33) (Figure S4b).

A joint analysis of gut metabolites and bacterial communities was performed, as described by Singh and colleagues [64]. The resulting combined dataset consists of two matrices of the 70 abundant ASVs, *i.e.*, representing at least 1% of reads in at least one sample, and the 1674 metabolites, sufficiently well correlated together (76%) to explore associations between ASVs and metabolites. The relevance network illustrates correlations between the most highly associated ASVs and metabolites discriminating among the five treatments. Four ASVs (ASV1349, ASV1564, ASV475, ASV1995) appear negatively correlated with several metabolites and are the least abundant in d28_0 (Fig. 2e and Fig. 4b). Differently, the two other ASVs, namely ASV1543 (*Epulopiscium*) and ASV2350 (Barnesiellaceae), are positively correlated with few metabolites and are the most abundant in d28_0 (Fig. 4b). Unfortunately, due to the general lack of proper metabolite databases [77], only few of the metabolites presenting high correlation with abundant ASVs could be annotated, and these mainly corresponded to amino acids or small peptides (Dataset S5).

### Diversity and composition of the gut bacterial microbiome and metabolome after depuration (d33) and a second exposure (d39)

**Fig. 5:**
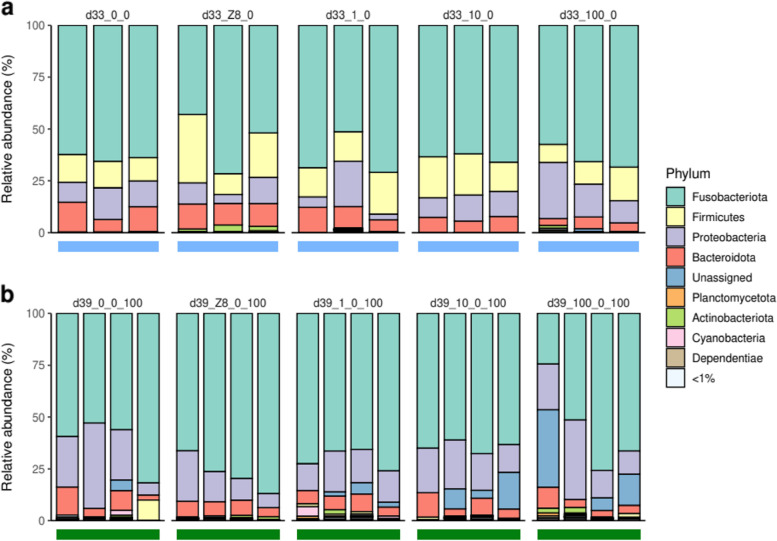
Depuration and a second *Microcystis* bloom impact the gut microbiota community. Relative abundance of gut bacterial phyla at d33, after 4 days in water following the d28 treatment (**a**), and at d39 after 5 additional days exposed to *M. aeruginosa* (**b**). Colored horizontal bars represent the different imposed treatments: blue = water, dark green = 100 µg.L^−1^ Chl*a*. Individuals sampled from a given aquarium (blue then green box below histograms) are organized in columns

After d28, fish from all 5 treatments were transferred to clean water (Fig. 1). At d33, some specimens were sampled, while others were transferred to a second exposure to *M. aeruginosa* (100 µg.L^−1^ Chl*a*), then sampled at d39. The species richness of gut-associated communities decreases between d28 and d33 (over 82 versus 50 ± 24 ASVs), then increases at d39 (76 ± 22 ASV). The PCoA discriminates between gut communities from fish exposed to 100 µg.L^−1^ Chl*a* (d28_100 and d39) and other treatments (Figure S5a). Community compositions differ among treatments (PERMANOVA, weighted UniFrac, *p* < 0.001). Treatment d33 differs from all other treatments (*p* < 0.05) except for d28_0 (*p* = 0.063); in other words, bacterial communities at d33 are mostly similar to those observed in the d28_0 treatment. Interestingly, *Microcystis*-affiliated ASVs are almost absent (below 0.2% of reads) from the water and absent from gut samples at d33. Communities at d39 differ from all other treatments (*p* < 0.05), except for d28_100 (*p* = 0.063); indicating that bacterial communities at d39 are similar to those from the d28 treatment exposed to the highest bloom intensity. Interestingly, *Microcystis*-affiliated ASVs are abundant at d39 in the water (9.8 to 18.1% of reads) and occur in the gut samples (up to 4.6%). Variance differs among treatments (PERMDISP, *p* < 0.01), particularly between d33 and all other treatments (*p* < 0.05), and between d39 and treatments d33 and d28_Z8 (*p* < 0.05), with d33 and d39 displaying lower variance. Some phyla are present in similar abundances at d33 and d39, including Fusobacteriota (62 ± 8% and 66 ± 14%, respectively), Proteobacteria (12 ± 6% and 19 ± 9%) and Bacteroidota (9 ± 3% and 7 ± 3%, Fig. 5a, b). Firmicutes, again mostly consisting of ASV1662, are present at d33 and almost absent at d39 (15.4 ± 6.4% versus 0.8 ± 1.8%). Other significant changes have been observed in specific ASVs between d33 and d39 (Figure S6a) among which seven were already observed to vary between the different d28 treatments (Fig. 2e and Figure S2a). Interestingly, ASV1620 (*Cetobacterium*), that was found abundant and stable among the different d28 treatments, is still remarkably stable at d33 and d39 (58.8 ± 7.4% and 61.7 ± 13.2%).

The gut metabolite profiles are significantly different among treatments (PERMANOVA, euclidean distance, *p* < 0.001, Figure S5b), especially between d33 and all treatments (*p* < 0.05) except d28_Z8 and d28_1, as well as between d39 and all others (*p* < 0.05) except d28_Z8 and d28_10. Contrary to bacterial microbiota composition, the d33 metabolomes are overall different from d28_0, and those of d39 are different from d28_100. A good correlation (81%) was observed between the gut ASVs and the metabolites discriminating between d33 and d39 when investigating the two matrices, containing 31 abundant ASVs and 1674 metabolites, respectively. The relevance network notably revealed two ASVs, belonging to genus *Vibrio* (ASV1406, ASV2161), that are negatively correlated with numerous metabolites, while two ASVs, belonging to genus *Reyranella* (ASV1030, ASV2363) appear positively correlated with numerous metabolites (Figure S6b). No differences occur among treatments in liver samples (*p* > 0.05, Figure S5c). In muscles, metabolomes at d33, d39, and d28_100 appear similar (*p* > 0.10), but different from all other treatment groups (Figure S5d).

### Occurrence of dominant ASVs in compartments other than gut

Dominant ASVs were searched for in fish food, *M. aeruginosa* culture, water, biofilm, and faeces (Figure S7). ASV1620 (*Cetobacterium*) is most abundant in guts (44.3 ± 20.4%) then in faeces (28.6 ± 15.3%), congruent with its best matches in the database which are fish gastrointestinal bacteria (zebrafish, Nile Tilapia). ASV1349 (*Flavobacterium*) and ASV1564 (*Aeromonas*) are most abundant in faeces (8.8 ± 9.6% and 7.8 ± 2.8%, respectively), then guts (2.1 ± 4.4% and 3.4 ± 2.6%, respectively) and biofilms (2.0 ± 3.3% and 3.4 ± 2.5%, respectively). This is congruent with their respective BLASTN hits with bacteria from fish intestinal tracts or aquatic environments. ASV1644 (unassigned Bacteria) is also most abundant in faeces (10.7 ± 7.4%), then in guts (3.7 ± 6.6%), but could not be assigned taxonomically. The Firmicutes ASV1662 (*ZOR0006*) mainly occurs in guts (19.3 ± 16.9%) and is slightly abundant in faeces (1.2 ± 1.4%), in accordance with the habitat of its closest matches, namely fish intestinal bacteria. Finally, ASV2363 (*Reyranella*) is most abundant in biofilms (4.4 ± 8.7%) compared to other compartments (water 2.2 ± 6.3%; feces 1.3 ± 2.7%; guts 1.2 ± 4.1%), and is related to various environmental bacteria.

## Discussion

Results from the 28-day exposure indicate that *Microcystis aeruginosa* blooms modify the fish gut bacterial microbiota compositions and have different effects on different taxa. Firmicutes, a phylum commonly found in gut bacterial communities of vertebrates and very likely implied in host metabolism processes [78–80], appear particularly sensitive as they decrease sharply upon exposure to the highest bloom intensity. Firmicutes were largely represented by a single bacterium (Erysipelotrichaceae_*ZOR0006*) that was almost absent outside of gut samples, suggesting being an indigenous and resident symbiont of *O. latipes* gut [81]. Kaakoush and colleagues [82] have previously discussed the central role of Erysipelotrichaceae in host metabolism and health in relation to diet specificity. According to our results based on the functional classification proposed by Vieira-Silva, *ZOR0006* displays a limited repertoire of metabolic modules compared to other bins such as *Cetobacterium* (16 vs. 54 GMM, respectively). Furthermore, most of the functions present in *ZOR0006* are also encoded by other bacteria in the microbiome, with the notable exception of the lactate-pyruvate interconversion module, a rare yet potentially important function otherwise only found in *Cetobacterium*. Indeed, lactate homeostasis is thought to be essential in the gut [83]. Although high concentrations are associated with inflammatory bowel disease in humans [84, 85], lactate has been shown to inhibit the growth of pathogenic bacteria [86], whereas pyruvate stimulates it [87]. In addition, lactate has recently been shown to be involved in the repair of the gut epithelium [88]. Symbiont abundance changes could thus affect the gut epithelium function as a barrier. Interestingly, the degradation activities of *ZOR0006*, like those of the other two bacteria whose abundance decreased after exposure, are totally oriented towards carbohydrates, suggesting a specialized degradation metabolism. The *ZOR0006* genus has already been observed to decrease in fish gut microbiota after exposing zebrafish to high concentrations of antibiotics or fungicides [89, 90]. Interestingly, the drop of *ZOR0006* abundance observed in this study occurs at a level between 10 and 100 µg.L^−1^ Chl*a.* Together with the observation of the greater influence of the highest bloom condition on the whole gut community, this suggests the existence of a cyanobacterial bloom threshold above which the gut microbiota composition is particularly altered. On the other hand, some abundant gut bacteria appear stable throughout the bloom, and even during the depuration and second exposure, the most remarkable being the bacterium affiliated to *Cetobacterium* (Fusobacteria). This genus is generally associated with healthy fish microbiota and notably contributes to host health as a cobalamin (vitamin B12) producer [91–93]. In addition to the confirmation of cobalamin biosynthesis ability, our analysis proved *Cetobacterium* to possess the largest set of GMM, a functional feature that could suggest a highly significant role as a symbiont. *Cetobacterium* is most likely a fish gut resident and has previously been reported stable in medaka fish upon exposure to pure microcystin-LR and cell extract of the *Microcystis* strain [35], supporting its maintenance through the exposure to cyanobacterial bloom and respective metabolites. Finally, relative abundances of some other bacteria increase during the *Microcystis* bloom*.* These could include transient gut bacteria that originate from the environment and proliferate once established in the gut, one example being *Reyranella* which also occurs in biofilms. These may also include rare gut resident taxa that can take advantage of peculiar conditions to proliferate. In our study, some of these bacteria, corresponding to *Flavobacterium*, *Aeromonas*, and *Shewanella*, are common inhabitants of fish guts or the environment, or potential fish gut pathogens according to the literature [94–96]. A similar increase of opportunistic bacteria was recently documented in guts of zebrafish exposed 96h to *M. aeruginosa* [10]. As previously shown for several metabolite mixtures from cyanobacterial cell extracts [35], exposure to whole cyanobacterial cells thus has a major impact on gut community compositions, indicating that fish microbiota might be impacted during the bloom as well as after bloom senescence which causes the release of the cyanobacterial cell contents into the surrounding water [97]. However, only the highest concentration of the bloom and/or its cell extract, that was explored in the present study, could induce most evident microbiota changes, suggesting that the microbiota responsiveness might be dose dependent. The cyanobacterial strain PMC 728.11 contains various secondary metabolites that may be responsible for variations in fish gut microbiota. Among those already identified and specifically those potentially produced by the strain [35, 98], cyanopeptides, such as aerucyclamides and bacteriocins, are thought to exhibit potent antimicrobial or cytotoxic bioactivities [99, 100], and could directly impact the microbiota during *Microcystis* cell digestion into the intestine lumen.

Metagenome-based investigation confirms the variations of taxa abundances, but very few taxa-specific known (*i.e.*, annotated) functions were identified for the bacteria that displayed the highest abundance variations, including for the Firmicutes *ZOR0006* which displays the smallest set of GMM. This is at first consistent with the common claim that a change in microbiota composition does not necessarily imply a change in the functions as estimated by gene content [101, 102]. However, similar genes are not necessarily expressed in the same way in different bacterial taxa, and thus phenotypes might still change dramatically despite the potential occurrence of similar gene contents. Indeed, variations in metabolite profiles reveal obvious functional variations induced by the different treatments, in particular changes in gut metabolite composition associated with increasing bloom concentrations. These changes could thus be related to unannotated COGs, much more numerous among the differentially abundant bacteria compared to annotated KO, or could result from variations in the expression of metabolic pathways in the holobiont. Whatsoever, the correlation observed between some dominant bacteria and many metabolites supports that the response of gut microbiota composition and that of the holobiont’s gut metabolome are linked. Gut community disruption is associated with metabolic changes, especially in the presence of higher bloom levels. This could lead to a potential dysbiotic state, however defining eubiotic versus dysbsiotic state is not straightforward, as discussed elsewhere [103, 104], and will require exploring various additional holobiont physiological variables. Altogether, the crosstalk between gut bacteria and metabolites remains difficult to characterize. For example, the presence of pathogens enhanced by bloom-induced toxicological impairs of fish physiology could secondarily affect the holobiont gut metabolism. Alternatively, the variation of the quantity of some gut metabolites could allow the development of opportunistic pathogens, as reported in mammals [105, 106]. Interestingly, variations of metabolite composition in muscles and livers remains limited in comparison to those observed in the gut, even after 28 days, implying that the gut metabolome compartment is more responsive to the *Microcystis aeruginosa* exposure. This observation is congruent with the fact that the gut is more directly exposed to the surrounding environment, through ingestion of water that contains various organisms and their associated compounds, and emphasizes the relevance of investigating the gut microbiota, as the gut epithelium sits at the interface between the host and its environment [32, 33]. Changes in microbiome and overall holobiont functions seem to be deeply linked, despite that the respective contribution of the host and the bacteria community cannot yet be disentangled due to the current impossibility to link a metabolite to its organism of origin within the holobiont and will require further dedicated functional analysis [107].

After the first 28-day bloom, depuration in clear water seems to have restored, to a certain extent, gut microbiota compositions. Indeed, compositions at d33 tended to resemble those of naïve specimens, not previously exposed to *M. aeruginosa* or to Z8 (d28_0). This indicates that the gut community composition is resilient, even over a relatively short time. The following second high-intensity bloom on the other hand yielded bacterial communities resembling those of specimens exposed to the first high-intensity bloom (d28_100), including the disappearance of Firmicutes and the stability of *Cetobacterium*. This indicates that a short duration bloom (5 days) already has a strong effect [37]. Gut communities thus quickly respond to the presence or absence of *M. aeruginosa* and associated bioactive compounds. Effects of the first and the second bloom are however not strictly identical. Firmicutes for example show reproducible behavior, disappearing upon first and second bloom, and seem to quickly re-establish post-bloom, suggesting they are not resistant, but remarkably resilient. However, the relative abundance of other taxa (including *Flavobacterium*, *Reyranella*, *Shewanella*), whose abundance increased upon the 28 days bloom, did not increase during the second 5-day exposure. It remains here difficult to compare variations of most non-dominant bacteria between d33 and d39 because of the substantial inter-individual variation of microbiota composition, combined with the limited sample size in terms of individual number per condition, compared to d28 conditions. Stress associated with specimen handling, the transfer of fish to newly cleaned aquaria after 28 days, has previously been referenced as a moderate, but possible, source of stress [108] and also could be involved to a certain extent in some of the differences. Contrary to gut community compositions, the metabolite composition in fish guts after the depuration and the second exposure did not tend towards those observed in unexposed and highest bloom at d28, respectively. So, metabolite compositions do not linearly follow the trends found in community compositions. This also suggests that the depuration and the second exposure may have been too prompt to induce major shifts in the gut metabolome, as these compartments may present different kinetics. Thus, the dynamics of gut microbiota and metabolome are very likely not identical, and the microbiota composition may somehow respond to changes faster than the metabolome. This would suggest that longer blooms occurring in nature may have more effect and functional consequences for the holobiont homeostasis compared to shorter blooms [109, 110].

## Conclusions

Overall, this study emphasizes that cyanobacterial blooms have the potential to alter fish gut microbiota composition and holobiont functions. Additionally, the drop of Firmicutes abundance induced by *M. aeruginosa* bloom which threshold level would be comprised between 10 and 100 µg.L^−1^ Chl*a*, together with the observation of a greater influence of higher bloom condition (100) on the overall gut community, support the existence of a notable tipping point for the responsiveness of gut microbiome to cyanobacterial bloom intensity. This finding could have important eco(toxico)logical consequences, as these levels are commonly reached in natural ecosystems during typical *Microcystis* bloom episodes worldwide, suggesting that destabilization of fish gut communities might be a very common event [42]. In nature, cyanobacterial blooms are nowadays becoming increasingly frequent [3], and sometimes persistent among seasons. Freshwater fish, especially those living in shallow water ponds, face numerous bloom episodes of varying durations during their lifetime. Consequences of iterative (up to chronic) exposures on the holobiont should be explored. Indeed, successive blooms without enough recovery time could induce a cumulative drift of the gut microbiota and metabolome, leading to suboptimal states that may lead to host health impairment [111]. This phenomenon could be of major significance for fish health in eutrophic natural ecosystems as well as aquaculture ponds where cyanobacteria often proliferate. Testing the effects of a single live *M. aeruginosa* strain administrated by simple balneation on a model teleost fish is a first step towards understanding bloom effects on fish microbiota and holobiont health. In the future, particular attention should also be paid to the natural diversity of cyanobacterial within blooms, as successive blooms often involve different species that produce different metabolite cocktails [112].

## Supplementary Information

**Additional file 1:** **Supplementary Note 1.** Dataset pre-processing and bacterial community comparison within the entire sample set. **Supplementary Note 2.** Metabolic content of *Microcystis aeruginosa* cultures. **Figure S1.** (a-c) Alpha-diversity metrics (species richness, Shannon, evenness) in the different sample types. Means are represented by a red dot. (d) Principal coordinates analysis (PCoA) representing bacterial communities from the different compartments using the weighted UniFrac distance. **Figure S2. **(a-b) Differentially abundant fish gut bacteria between treatments d28_0 (blue) and d28_100 (dark green) based on 16S rRNA reads (a) and shotgun metagenome sequencing (b). The absence of 16S rRNA sequences in metagenome bins does not allow to directly connect ASVs with bins, however the coloured taxon names represent bacteria with the same taxonomic affiliation. Lowest assigned taxonomic levels are displayed with the level specified by a single letter. (a) Discriminant ASVs based on the Linear discriminant analysis (LDA) effect size (LEfSe) with a LDA score above 3.5 and their variations in relative abundance in fish guts. The coloured boxes represent the treatment where each ASV is found the most abundant. The dots represent average relative abundance, the lines spread over the range of observed values. Only dominant ASVs (see text), were considered and further investigated. (b) Taxonomic affiliations (using the BAT method) of bins displaying significantly different relative abundances based on the Wilcoxon ranksum test (*p* < 0.05) and their relative abundance across fish gut samples. The CAT method was used to affiliate bin10 (Rhodospirillales) at a lower taxonomic level, *i.e.* *Reyranella massiliensis*. The treatment where each bin is significantly more abundant is represented by the coloured box. (c) Overlap of KO counts among three groups: KO from bins significantly more abundant in d28_0 (blue) or in d28_100 (dark green) (*p* <0.05, Wilcoxon rank-sum test), or KO from bins non-significantly different between the two treatments (beige) (*p* > 0.05). **Figure S3.** Triplot representation of bin contributions in the degradation potential of the whole microbiome, defined as the fraction of GMM coded by a bin in each of the three major degradation types, amino acids, lipids and carbohydrates. Bin colors correspond to their abundance variation between experimental conditions. **Figure S4.** Principal component analyses (PCA) illustrating the metabolite composition in fish livers (a) and muscles (b) from the five different treatments during 28 days. **Figure S5.** Comparisons of the composition of bacterial communities (a) and metabolite profiles (b) in fish guts, and the composition of metabolite profiles between the three different sampled organs, guts (b), livers (c) and muscles (d). The three organs were either exposed long-term during 28 (d28) days (full circles or diamonds) or short-term during 4 (d33) or 5 (d39) days (open circles or diamonds). (a) Principal coordinates analysis (PCoA) on weighted UniFrac distance illustrating bacterial composition in fish guts. (b-d) Principal component analyses (PCA) representing metabolite profiles in fish guts, livers and muscles. **Figure S6.** (a) Significant ASVs from the LEfSe analysis, differentially abundant between d33 gut samples (in water) and d39 gut samples (in 100 µg.L^-1^ Chl*a*). The coloured boxes represent the treatment where ASVs are found more abundant. Dots and lines indicate mean and range of values, respectively. The displayed taxonomic affiliations correspond to the lowest assigned level, displayed using the first taxon letter. The two ASVs (ASV2363, ASV1662) underlined are also found differentially abundant between d28_0 and d28_100. (b) Relevance network analysis representing the most correlated ASVs and metabolites discriminating d33 and d39 gut samples. Only ASVs (in orange) and metabolites (in white) associated with Pearson correlation scores above ±0.714 are displayed. Coloured segments represent Pearson correlation values, either positive (red) or negative (blue). **Figure S7.** Relative abundance of dominant ASVs across compartments other than gut (culture, water, biofilm, gut and faeces). ASVs were searched for in the fish food sample discarded at the rarefaction step, but any ASVs were found (only 1% of reads for ASV1620). **Table S1.** Composition of annotated metabolites of the PMC 728.11 *Microcystis aeruginosa* strain. Percentages represent the proportions of each annotated cluster on the total annotated clusters. Abbreviation: AA = Amino Acids.

**Additional file 2:** **Dataset S1.** Sampling counts during the whole experiment.

**Additional file 3:** **Dataset S2.** Monitoring of abiotic and biotic parameters during the experiment.

**Additional file 4:** **Dataset S3.** Annotations of metabolites from the *Microcystis aeruginosa* strain PMC 728.11.

**Additional file 5:** **Dataset S4.** 16S rRNA gene sequencing and quality filtered read counts, SRA database accession numbers, and measures of alpha-diversity metrics (species richness, Shannon, evenness).

**Additional file 6:** **Dataset S5.** Annotations of the most correlated metabolites with bacteria from fish guts at day 28.

## Data Availability

16S rRNA gene sequencing and shotgun raw data are deposited into the GenBank SRA database under the BioProject PRJNA746242 (samples SAMN20080275 to SAMN20080438). The full R code and QIIME 2 script are available on Zenodo (10.5281/zenodo.5922616).
